# Utilizing group-based models to identify adverse event patterns after an intervention

**DOI:** 10.3389/fmed.2025.1637091

**Published:** 2025-09-16

**Authors:** Wei Wang, Sara Abbaspour, Kimberly G. Blumenthal, Dean M. Hashimoto, Gregory K. Robbins, Elizabeth B. Klerman

**Affiliations:** ^1^Division of Sleep and Circadian Disorders, Brigham and Women’s Hospital, Harvard Medical School, Boston, MA, United States; ^2^Division of Sleep, Department of Neurology, Massachusetts General Hospital, Boston, MA, United States; ^3^Division of Rheumatology, Allergy and Immunology, Department of Medicine, Massachusetts General Hospital, Harvard Medical School, Boston, MA, United States; ^4^Workplace Health, Mass General Brigham, Boston, MA, United States; ^5^Division of Infectious Diseases, Massachusetts General Hospital, Boston, MA, United States

**Keywords:** side-effects, adverse events, trajectory analyses, COVID-19 adverse effects monitoring, vaccines, adverse effects following immunization (AEFI)

## Abstract

**Background:**

Standard adverse event (AE) monitoring only records whether events occur after the intervention, and not whether these events vary over time.

**Objective:**

To test whether there were statistically distinct time-varying trajectories of AE (e.g., “side effects”) after an intervention and identify characteristics of individuals associated with these patterns.

**Design:**

Group-based trajectory models applied to an observational study of individuals who received one or two doses of a mRNA COVID-19 vaccine (i.e., the intervention).

**Participants:**

50,484 healthcare personnel who received their vaccinations within the Mass General Brigham (MGB) healthcare system.

**Interventions:**

Vaccination.

**Main measures:**

Allergic and non-allergic AE for 1–3 days after each of two COVID-19 vaccinations.

**Key results:**

Trajectories models identified distinct groups with different trajectories after intervention: two groups after the first vaccination and five groups after the second vaccination. These groups differed by demographics, age, prior prescription for epinephrine auto-injectors, prior COVID-19 history, time-of-day of vaccination, and vaccine manufacturer.

**Conclusion:**

Several different time-based trajectories after the intervention (e.g., first two COVID-19 vaccinations) were noted; individuals in these groups varied by demographic and clinical criteria. These time-based methods may be able to identify groups at higher risk of future adverse reactions, provide a basis for future studies of the physiology underlying these risk differentials, and improve counseling surrounding interventions associated with AEs. We suggest that trajectory-based methods be added to post-intervention surveillance.

## Introduction

Clinical signs and symptoms, including adverse events (e.g., “side effects,” adverse effects following immunization (AEFI)) from drugs, vaccines, and other interventions, may occur in clusters and vary over time. Standard adverse event monitoring, however, only records whether an event has occurred after the intervention (1). With access to longitudinal data from a large cohort of employees from a single healthcare system after their first and second doses of COVID-19 vaccinations, we could utilize time-varying group-based analytic models to define distinct groups of individuals with differing time-varying vaccine AEs. We hypothesized that different time-varying clusters would be found and that these clusters would have differential risk factors (e.g., sex, age, history of allergy). We propose that trajectory of symptoms could be useful for future AE studies after any intervention (e.g., drug, vaccine, surgery); different risk factors and trajectories over time could enable targeting physiological mechanisms, be used for patient and clinician education, and be used for patient planning (e.g., schedule intervention when fewer AEs are expected), which could improve compliance and reduce hesitancy for the intervention.

## Methods

In this observational study, data were collected from 50,484 healthcare personnel who received one or two doses of either the Pfizer or Moderna mRNA COVID-19 vaccine between December 2020 and April 2021 within the Mass General Brigham (MGB) healthcare system. Demographic data, allergy history, prior epinephrine auto-injector prescription, prior COVID-19 history, vaccine manufacturer (Pfizer or Moderna), and time-of-day of vaccination data were retrieved from the MGB electronic health record’s COVID-19 Datamart. Self-reported symptoms for 1–3 days after each vaccination were collected using a REDCap survey that was sent daily for 3 days after each vaccination. Detailed information about the REDCap AE survey has been previously reported (2, 3).

Group-based trajectory models (4) were utilized to identify patterns of participant-reported post-vaccination AEs over the 3 days following each vaccination dose. Participants exhibiting similar patterns of daily AEs were clustered into distinct subgroups using logistic modeling of dichotomous outcomes conditioned on group membership. The optimal number of groups was determined using Akaike (AIC) and Bayesian (BIC) information criteria. A second set of logistic regression models was then employed to explore the associations between these trajectory subgroups and potential predictors. Any symptoms of rash, hives, swelling, and wheezing were grouped together as any allergic-type AEs and coded as a binary outcome (yes, no). Symptoms such as new headache, fatigue, joint pain, muscle pain, and fever were grouped as “any non-allergic AE” and coded as (lower/higher severity, none). Allergic-type and non-allergic AEs were further combined into a single outcome: any allergic-type and/or any non-allergic AEs (yes or no). If on any day after vaccination, a participant did not respond to at least one of the 9 symptom questions (e.g., rash, hives, swelling, and wheezing, new headache, fatigue, joint pain, muscle pain, or fever), the AE outcome for that day was coded as “Missing.” Potential factors considered in the models included age group (18–40, 41–60; >60), gender, race/ethnicity groups (White/non-Hispanic, Non-white/non-Hispanic, Any race-Hispanic (“Hispanic”), Any race/other Ethnicity [including not reported]), epinephrine auto-injector prescription, vaccine manufacturer (Pfizer, Moderna), time-of-day of vaccination (6:00–10:59, 11:00–15:59, 16:00–21:59), prior allergy history (yes, no), and any prior COVID-19 diagnosis or positive test (yes, no). Adjusted Odds Ratios (ORs) of any AEs and their 95% confidence intervals (CIs) were reported for each predictor in the multivariable logistic regression models. We examined the relationships both after the first vaccination and the second vaccination doses. All trajectory analyses were repeated using two approaches: (1) using all available data, assuming data were missing at random (“Full”); and (2) using “Complete-case” subsets, where there was no missing data for all 3 days following the first vaccination dose and all 6 days following both doses (3 days after each dose). No imputation methods were used, as the longitudinal data patterns were complex and there were no reasonable assumptions about the missing data distribution that could have led to unreliable estimates and artificially inflated precision. All analyses were performed using SAS Version 9.4. Group-based trajectory analyses were conducted using PROC TRAJ.

These analyses were conducted under MGB Human Subjects (IRB) Protocol 2021P001080. The IRB waived the requirement for informed consent.

## Results

Data analysis of AEs after the first vaccination included 50,484 participants, of whom 34,803 had non-missing data for all three follow-up days. Among these, 50,270 participants received the same vaccine brand for both doses. The Complete-case subset for AEs following both doses consisted of 24,529 participants with no missing data across all six follow-up days. Results for the Full dataset (with missing data) are in the Supplementary material.

**Figure 1:**
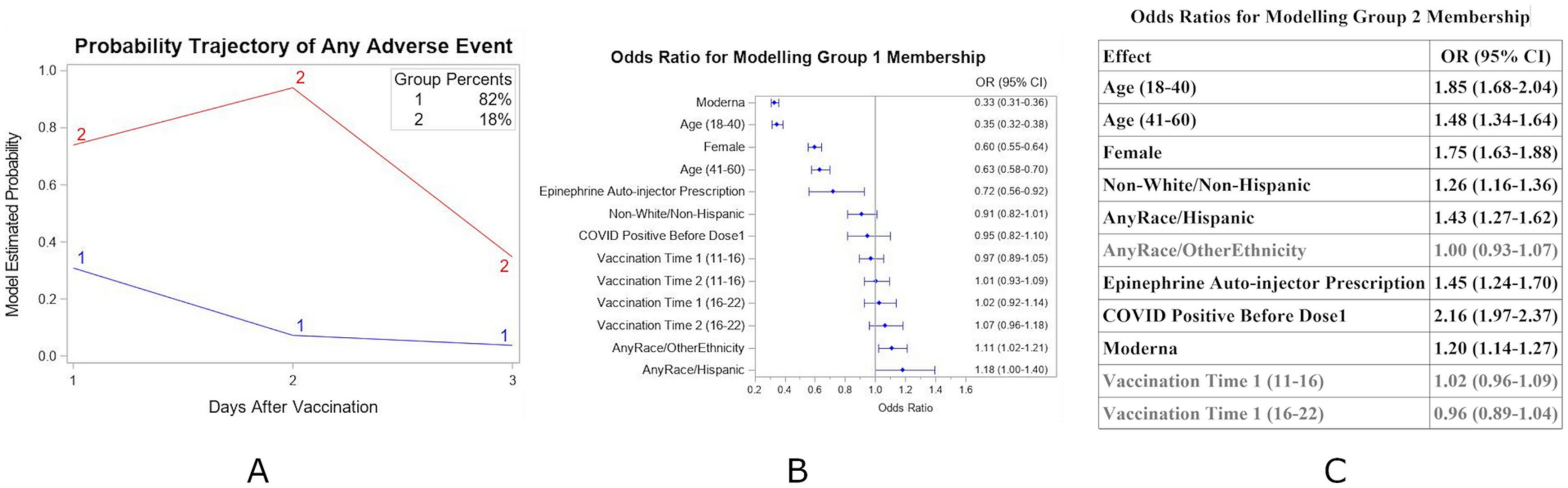
**(A)** Estimated probability and group membership using longitudinally self-reported AEs of any symptoms on days 1, 2, or 3 after vaccination dose 1 using the Complete-case dataset (*N* = 34,803). **(B)** Forest plot for the adjusted odds ratios of any AEs based on the multivariable binary logistic regression model for Group 1 membership. Reference values: no prior COVID positive diagnosis, age >60, Male, no Epinephrine Auto-injector, White/non-Hispanic race/ethnicity, Pfizer vaccine, and Vaccination time 6–11 am. **(C)** Adjusted odds ratios of any AEs based on the multivariable nominal logistic regression model for Group 2 membership using Group 1 as the reference group. Groups in bolded text were significant at *p* < 0.05. **(B,C)** Are complementary—with different reference groups.

After the first vaccination dose, two distinct groups were identified (Figure 1A) for the Complete-case subset. Group 1 members experienced minimal AEs only on the first day post-vaccination. In contrast, Group 2 members (18%) reported more severe symptoms on the second day but showed significant improvement by the third day. A forest plot for the adjusted odds ratios of any AEs based on the logistic regression model for the Group 1 membership is shown in Figure 1B. Participants with a history of prior COVID infection, those in the younger age groups (18–40 or 41–60), females, and those with a prior prescription for an Epinephrine Auto-injector had a higher likelihood of belonging in Group 2 (Figure 1C; note that Figures 1B,C are complementary since there are only two groups). Based on these results, individuals with the following characteristics would be more likely to have some symptoms on the first day after vaccination (i.e., Group 2 compared to Group 1), more symptoms on the second day, and fewer symptoms on day 3 than day 1: age 18–40 years (OR = 1.85, 95% CI = (1.68, 2.04)) or 41–60 years (OR = 1.48, 95% CI = (1.34, 1.64)), Female (OR = 1.75, 95% CI = (1.63, 1.88)), Non-White Non-Hispanic (OR = 1.26, 95% CI = (1.16, 1.36)) or Hispanic (OR = 1.43, 95% CI = (1.27, 1.62)), Epinephrine Auto-injector prescription (OR = 1.45, 95% CI = (1.24, 1.70)), history of COVID positive test before first vaccine dose (OR = 2.16, 95% CI = (1.97, 2.37)), and/or dosage with Moderna vaccine (OR = 1.20, 95% CI = (1.14, 1.27)).

**Figure 2:**
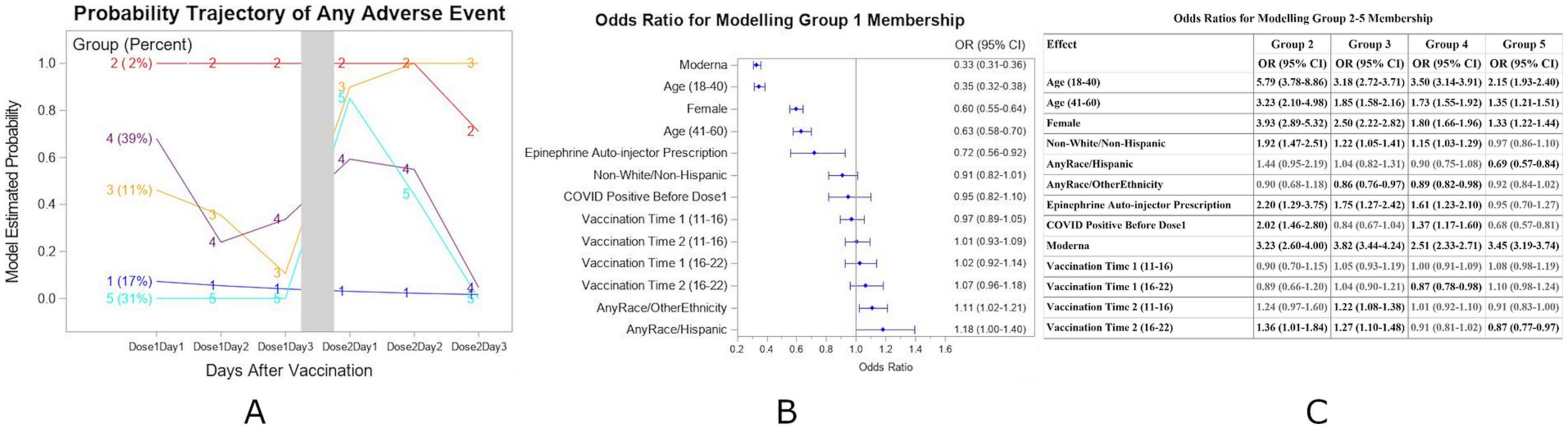
**(A)** Estimated probability and group membership using longitudinally self-reported AEs of any symptoms on days 1, 2, and 3 after each of vaccination doses 1 and 2 using the Complete-case dataset (*N* = 24,529). **(B)** Forest plot for the adjusted odds ratios of any AEs based on the multivariable binary logistic regression model for Group 1 membership. Reference values as in Figure 1. **(C)** Adjusted odds ratios of any AEs based on the multivariable nominal logistic regression model for Group 2–5 membership using Group 1 as the reference group. Groups in bolded text were significant at *p* < 0.05.

After the second vaccine dose, five distinct groups were identified based on the AEs reported (Figure 2A) for the Complete-case subset. Group 1 members experienced minimal AEs at all time points. A forest plot for the adjusted odds ratios of any AEs based on the multivariable binary logistic regression model for the Group1 membership is shown in Figure 2B. Group 2 members (2% of the participants) consistently experienced AEs after both doses. Group 3 members experienced some AEs after the first dose and significantly worse AEs after the second dose. Group 4 members reported AEs on the first day after each dose and improved thereafter. Group 5 members had minimal AEs after the first dose but experienced more AEs on the first day after the second dose. Individuals of younger age, female gender, and those who received the Moderna vaccine are associated with Group 2 (all *p* < 0.0001), showing the highest likelihood of experiencing AEs throughout all days following vaccinations. Adjusted odds ratios of any AEs based on the multivariable logistic regression model for Group 2–5 membership using Group 1 as the reference group are shown in Figure 2C.

In this analysis of data from both doses, individuals in Group 1 were more likely to report having no symptoms after both vaccinations. Group 1 characteristics included age over 60 years, Male, Hispanic, no Epinephrine Auto-injector prescription, and/or dosage with Pfizer vaccine. Compared with Group 1 individuals, those more likely to have symptoms on all 6 days of symptom monitoring (days 1–3 after each dose) (Group 2) were more likely to have the following characteristics: ages 18–40 (OR = 5.79, 95% CI = (3.78, 8.86)) or 41–60 (OR = 3.23, 95% CI = (2.10, 4.98)) years, Female (OR = 3.93, 95% CI = (2.89, 5.32)), Non-White/Non-Hispanic (OR = 1.92, 95% CI = (1.47, 2.51)), Vaccine dose 2 time 4–10 pm (OR = 1.36, 95% CI = (1.01, 1.84)), Epinephrine Auto-Injector Prescription (OR = 2.20, 95% CI = (1.20, 3.75), COVID Positive before Dose 1 (OR = 2.02, 95% CI (1.45, 2.80)) and/or dosage with Moderna vaccine (OR = 3.23, 95% CI = (2.60, 4.00)). For individuals with time varying probability of AEs (Groups 3, 4, 5), the different characteristics were Race and Ethnicity, history of COVID positivity before Dose 1, and/or vaccine time.

These patterns remained similar for most subgroups in analyses using the Full dataset (see Supplementary material).

## Discussion

We identified multiple groupings of time-based symptom trajectories after COVID-19 vaccination (i.e., an intervention) with different demographic and/or clinical characteristics for the groups. Our work is innovative in demonstrating that the *multiple* time-scale-based trajectory analyses yield additional information that may be clinically actionable, including (i) identifying those individuals with little risk of AE and those at higher risk – and the times of that increased risk—who might benefit from additional counseling or monitoring, and/or (ii) counseling individuals about when symptoms/signs may appear or lessen. The method applies to both allergic and non-allergic reactions, an area currently underrepresented in the literature, highlighting the need for such versatility in drug and vaccine safety, quality control, and patient compliance. Group-based trajectory methods provide an innovative way to monitor daily AEs across multiple doses of a drug or vaccine. One study of weight loss after medication in children performed trajectory analyses and found changes related to the drug and age of the child (5). Another study used trajectory analyses to monitor compliance with medication use (6). A study of response to multiple COVID-19 vaccines used a similar latent class analysis to compare adverse events occurring any time after each vaccine (7), not the time course of adverse events following each vaccine. Collection of the data needed for group-based trajectory analyses can be done semi-automatically using email links, text messages, and phone apps. This study had high response rates among healthcare providers (2); another study reported high response rates by patients who received automated text message for monitoring adverse events after immunization (8).

## Limitations

There are multiple limitations to this first demonstration of this methodology: (i) The AEs data were collected from an observational trial. Future work should include trials with appropriate randomization. (ii) The cohort consisted only of health care employees in the northeastern US, and thus the study findings related to specific AE trajectories may not be generalizable to other populations. Study populations may vary in reporting bias; highly motivated individuals may be more likely to report positive, negative, or no outcomes. Future work should be conducted in other populations. (iii) The data were collected after response to one potential intervention; future work should be conducted with other interventions (e.g., medications, medical devices, surgery).

## Conclusion

By analyzing trajectory patterns over time (rather than only the presence/absence of an adverse event at a fixed time), these methods may identify groups at higher risk of future adverse reactions, provide additional physiological information that may be useful for future basic and clinical studies of AEs, and improve counseling surrounding interventions associated with AEs. This approach allows for real-world post-marketing monitoring to track the evolution of AEs over time for single or multiple interventions (e.g., chemotherapy). Particularly useful in unpredictable, idiosyncratic reactions (in addition to relatively predictable and/or allergic reactions), this work may help optimize the timing of medications, vaccinations, or other interventions and serve as a versatile tool for pharmaceutical trial design, medication administration, and patient safety.

## Data Availability

The data analyzed in this study is subject to the following licenses/restrictions: Raw data will not be shared because they were originally collected from healthcare personnel for monitoring post-vaccination. Requests to access these datasets should be directed to ebklerman@mgh.harvard.edu.

## References

[1] Suku CK Hill G Sabblah G Darko M Muthuri G Abwao E. Experiences and lessons from implementing cohort event monitoring programmes for antimalarials in four African countries: results of a questionnaire-based survey. Drug Saf. (2015) 38:1115–26. doi: 10.1007/s40264-015-0331-7, PMID: 26267842 PMC4608977

[2] Shenoy ES Wickner PG West LR Banerji A Blumenthal KG Centi AJ. Symptom monitoring after coronavirus disease 2019 (COVID-19) vaccination in a large integrated healthcare system: separating symptoms from severe acute respiratory coronavirus virus 2 (SARS-CoV-2) infection. Infect Control Hosp Epidemiol. (2021) 43:1439–46. doi: 10.1017/ice.2021.449, PMID: 34726142 PMC8564030

[3] Abbaspour S Robbins GK Blumenthal KG Hashimoto D Hopcia K Mukerji SS. Identifying modifiable predictors of COVID-19 vaccine side effects: a machine learning approach. Vaccine. (2022) 10:1747. doi: 10.3390/vaccines10101747, PMID: 36298612 PMC9608090

[4] Nagin DS Jones BL Passos VL Tremblay RE. Group-based multi-trajectory modeling. Stat Methods Med Res. (2018) 27:2015–23. doi: 10.1177/0962280216673085, PMID: 29846144

[5] Pozzi M Pisano S Marano G Carnovale C Bravaccio C Rafaniello C. Weight-change trajectories of pediatric outpatients treated with risperidone or aripiprazole in a naturalistic setting. J Child Adolesc Psychopharmacol. (2019) 29:133–40. doi: 10.1089/cap.2018.0092, PMID: 30452281

[6] Frank AS Lupattelli A Matteson DS Nordeng H. Maternal use of thyroid hormone replacement therapy before, during, and after pregnancy: agreement between self-report and prescription records and group-based trajectory modeling of prescription patterns. CLEP. (2018) 10:1801–16. doi: 10.2147/CLEP.S175616, PMID: 30584374 PMC6283256

[7] Yamamoto C Kobashi Y Kawamura T Nishikawa Y Saito H Oguro F. Group of longitudinal adverse event patterns after the fourth dose of COVID-19 vaccination with a latent class analysis. Front Public Health. (2024) 12:1406315. doi: 10.3389/fpubh.2024.1406315, PMID: 39139673 PMC11320210

[8] Leeb A Regan AK Peters IJ Leeb C Leeb G Effler PV. Using automated text messages to monitor adverse events following immunisation in general practice. Med J Aust. (2014) 200:416–8. doi: 10.5694/mja13.11166, PMID: 24794676

