# Supplementary material for: Utilizing group-based models to identify adverse event patterns after an intervention
Source: Front Med (Lausanne). 2025 Sep 16;12:1637091. doi: 10.3389/fmed.2025.1637091 (PMC12479506; doi:10.3389/fmed.2025.1637091)
Supplement: Supplementary file 1 [file Data_Sheet_1.docx]

# Title: Utilizing Group-Based Models to Identify Adverse Event Patterns After an Intervention

Authors: Wei Wang PhD, Sara Abbaspour, Kimberly G Blumenthal MD MSC, Dean M Hashimoto MD MPH, Gregory K Robbins MD, Elizabeth B Klerman MD PhD

# Supplemental Material:

Results of the Full (with missing data) dataset. Main text contains results from Complete-case dataset.

**
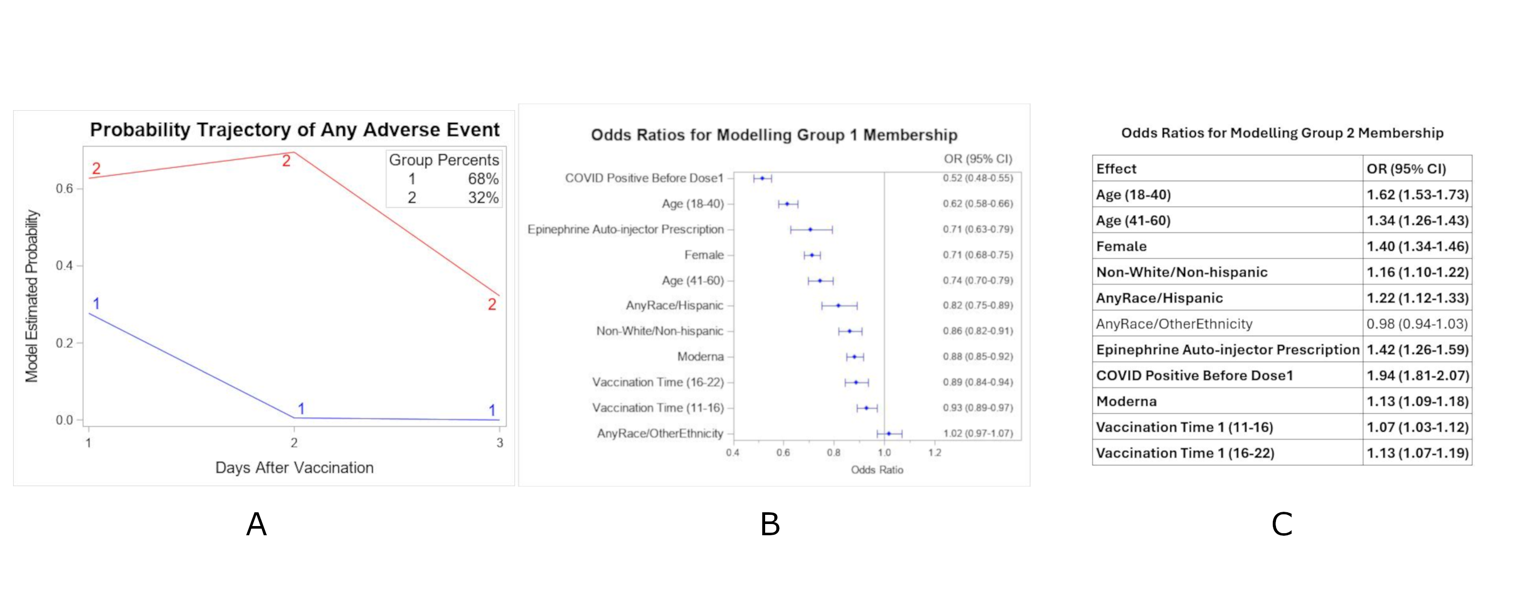
Supplementary Figure 1:** (A) Estimated probability and group membership using longitudinally self-reported AEs of any symptoms on days 1, 2, or 3 after vaccination dose 1 using the Full dataset (N=50,484). (B) Forest plot for the adjusted odds ratios of any AEs based on the multivariable binary logistic regression model for Group 1 membership. Reference values: no prior COVID positive diagnosis, age >60, Male, no Epinephrine Auto-injector, White/non-Hispanic race/ethnicity, Pfizer vaccine, and Vaccination time 7-10 am. (C) Adjusted odds ratios of any AEs based on the multivariable nominal logistic regression model for Group 2 membership using Group 1 as the reference group. Groups in bolded text were significant at p< 0.05. Note that (B) and (C) are complementary - with different reference groups. Missing data for AE outcomes occurred in 6,707 (13.3%), 7,180 (14.2%), and 8,182 (16.2%) participants on days 1, 2, and 3, respectively.

**
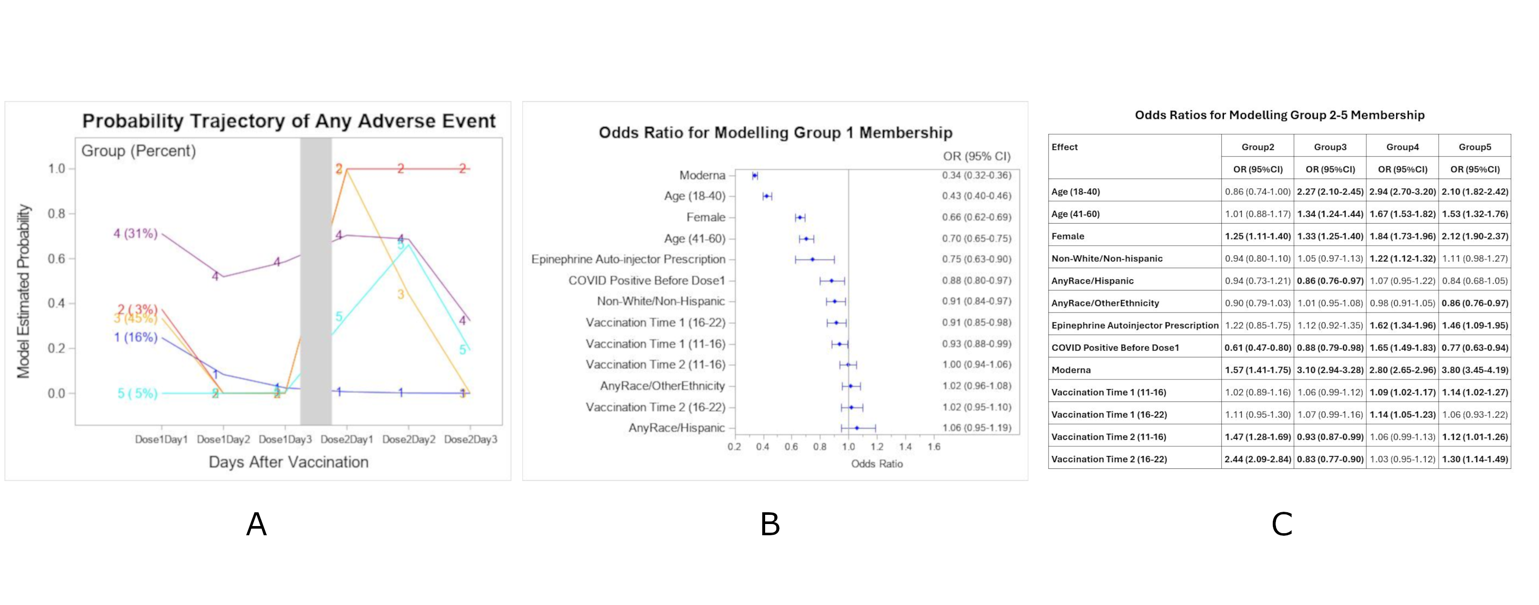
Supplementary Figure 2:** (A) Estimated probability and group membership using longitudinally self-reported AEs of any symptoms on days 1, 2, and 3 after each of vaccination doses 1 and 2 using the Full dataset (N=50,270). (B) Forest plot for the adjusted odds ratios of any AEs based on the multivariable binary logistic regression model for Group 1 membership. Reference values as in Figure 1. (c) Adjusted odds ratios of any AEs based on the multivariable nominal logistic regression model for Group 2-5 membership using Group 1 as the reference group. Groups in **bolded text** were significant at p< 0.05. Missing data for AE outcomes occurred in 6,664 (13.3%), 7,136 (14.2%), 8,117 (16.2%), 11,600 (23.1%), 12,937 (25.7%), and 15,196 (30.2%) missing data points for days 1, 2, and 3, respectively.
